# Supplementary material for: Robustness of Physiological Synchrony in Wearable Electrodermal Activity and Heart Rate as a Measure of Attentional Engagement to Movie Clips
Source: Sensors (Basel). 2023 Mar 10;23(6):3006. doi: 10.3390/s23063006 (PMC10058467; doi:10.3390/s23063006)
Supplement: Supplementary file 1 [file sensors-23-03006-s001.zip › sensors-2158980-supplementary.pdf]

Supplementary material

Corresponding to: Stuldreher et al.: Robustness of physiological synchrony in wearable electrodermal activity and heart rate as a measure of attentional engagement to movie clips

Table S1. Correlations between number of correct answers on questions about the content of the movie and PVT performance measures for participants in the TA condition.

Movie	Movie vs. PVT reaction time
Chauffeur	$r = 0.53, p = .467$
El Mourabbi	$r = -0.28, p = .437$
De Chinese Muur	$r = -0.62, p = .244$
One of the boys	$r = -0.59, p = .058$
Samual	$r = -0.20, p = .672$
Turn it around	$r = -0.46, p = .231$
Overall:	$\boldsymbol{r = -0.39, p = .007}$

Table S2. Questions and answers about the content of the six movie clips.

El Mourrabi

- Wat is de naam van deze film?
  El Mourabi
- Is de pizzabezorger blij met zijn fooi?
  Nee, hij scheldt de fooigever stiekem uit
- Hoe heet de pizzabezorger?
  Rachid
- Op wat voor kleur scooter rijdt de bezorger?
  Rood
- Welke kleur kleren heeft de baby?
  Grijs-blauw gestreept
- Waar verstopt Rachid de baby voor zijn vrienden en collega's buiten het pizzarestaurant?
  In de pizzadoos van zijn scooter
- Waarom zet de pizzabezorger de muziek op zijn kamer zo hard?
  Om de huilende baby te maskeren
- Hoe heet het pizzarestaurant?
  Pizza Galeria
- Waar geeft Rachid de baby af?
  Buiten bij het ziekenhuis
- Welke dieren bekeken de pizzabezorger en baby?
  Geiten / schapen

Samual

- Wat is de naam van deze film?
  Samual
- Hoe komt Samual in eerste instantie aan geld?
  Bedelen op straat.
- Wat doet Samual met het muntgeld?
  Hij probeert te bellen bij een telefooncel

- Waarom is Samual in Amsterdam?
  Haar dochter woont tegenwoordig in Amsterdam en is zwanger.
- Waar zijn volgens Samual de dromen van de toekomst?
  Aan de overkant van de zee
- Aan wie wilt Samual een briefje gevem bij vertrek uit het politiebureau?
  Het meisje in het kinderdagverblijf
- Aan wie doet het meisje in het kinderdagverblijf Samual denken?
  Aan zijn eigen dochter.
- Hoe maakt de geldwisselaar duidelijk dat hij geen geld kan wisselen?
  Door zijn hoofd te schudden
- Wat staat er op het briefje dat Samual bij zich had?
  Een telefoonnummer
- Wat staat er op het raam van het kinderdagverblijf geschilderd?
  Een konijn

Turn it around

- Wat is de naam van deze film?
  Turn it around
- Wie is ‘het lekkerste wijf op het feest’?
  Denise
- Aan wie wordt hoofdpersoon Bram op zijn verzoek voorgesteld?
  Floor / Florian
- Waar komt Florian vandaan?
  Amsterdam
- Wat is de reden dat Bram en Florian naar buiten gaan?
  Binnen is het te druk
- Bij welke term schrikt bram terwijl zijn hand dichter naar die van Floor kruipt?
  Vuile flikker
- Waarom kan bram niet mee met het festival waar Floor ook heen gaat?
  Er zijn geen kaarten meer beschikbaar
- Welk spel speelt de groep in deze film?
  Flesje draaien
- Waarom wilt Bram tegen het einde van de film nog flesje draaien?
  Omdat floor naar huis gaat en Bram het liefste Floor nog wilt zoenen.
- Komt het flesje van Bram uit bij Floor?
  Nee
- Wat is de kleur van het etiket op het flesje?
  Blauw

De Chinese muur

- Wat bestelt de vrouw in het begin van de film?
  Tomatensoep met kip, met veel kip.
- Aan wie doet de man, die aan een van de andere tafels zit, de vrouwelijke hoofdpersoon aan denken?
  Fred
- In welke ijs heeft de vrouwelijke hoofdpersoon zin?
  Dame Blanche
- Waarom zingt iedereen in het restaurant voor de vrouwelijke hoofdpersoon?

Omdat ze zei dat ze jarig was, terwijl ze dat eigenlijk niet is.

- Waarom is het niet gek dat de personen tegenover de vrouwelijke hoofdpersoon op elkaar lijken?
  Het zijn broer en zus
- Hoe komen de gasten in het restaurant over op de persoon die op het einde binnenkomt om Chinees af te halen?
  Als een grote happy family.
- Hoeveel sterretjes stonden er op de taart?
  Vier
- Wat was er aan de hand met de vrouw van Fred?
  Ze was dement
- Hoe heet de zoon van de vrouwelijke hoofdpersoon?
  Robert
- Hoe dacht de vrouwelijke hoofdpersoon dat de groep jonge mensen elkaar zou kennen, en wat was dat daadwerkelijke?
  Iets met computers; schaatsclub

One of the boys

- Wat is volgens de hoofdrolspeler een wet van de natuur?
  'Tijdens de zomer worden alle chicks lekkerder'
- Hoe heet de hoofdrolspeler?
  Daan
- Waarom wordt de hoofdrolspeler boos en agressief?
  Omdat zijn mannelijke teamgenoten Elisabeth lastigvallen.
- Wat doet Daan wanneer hij samen met Elisabeth in de kleedkamer is?
  Hij geeft haar een kus
- Welke sport speelt het team in deze film?
  Korfbal
- Wat voor fruit ligt er in de kleedkamer?
  Banaan
- Waar is de hoofdpersoon op vakantie geweest?
  Drenthe
- Wat staat er op het briefje dat aan de muur hangt in de kleedkamer?
  Is het ballenhok dicht
- Welke kleur heeft de bal?
  Geel-blauw
- Welke kleur is het shirt dat de gezette jongen draagt?
  Roze

Chauffeur

- Wat is de kleur van de dienstauto van de chauffeur?
  Zwart
- Wat vraagt de chauffeur aan de twee kinderen die vlak bij zijn auto staan op het schoolplein?
  Of ze 'Tom' kennen en hem willen halen.
- Hoe heet de jongen die met de chauffeur mee gaat?
  Tom
- Wat belooft de chauffeur de jongen als hij mee gaat?
  Een modelauto
- Wat doen de chauffeur en de jongen in de auto op het afgelegen terrein?

De jongen rijdt zelf een stuk

- Wat doen de chauffeur en de jongen nadat de jongen zelf een stukje heeft gereden?
  Eten halen bij de McDonalds
- Wat bestelt de jongen bij het restaurant?
  Een happymeal en een vanille milkshake.
- Wat is de relatie tussen de jongen en de chauffeur?
  Het kind is de zoon van de chauffeur.
- Waarom wordt de chauffeur boos en zegt hij ‘godverdomme’ tegen de jongen in de auto?
  Het kind morst zijn milkshake.
- Wat zegt de chauffeur tegen de moeder van de jongen?
  Helemaal niks

ISC HR

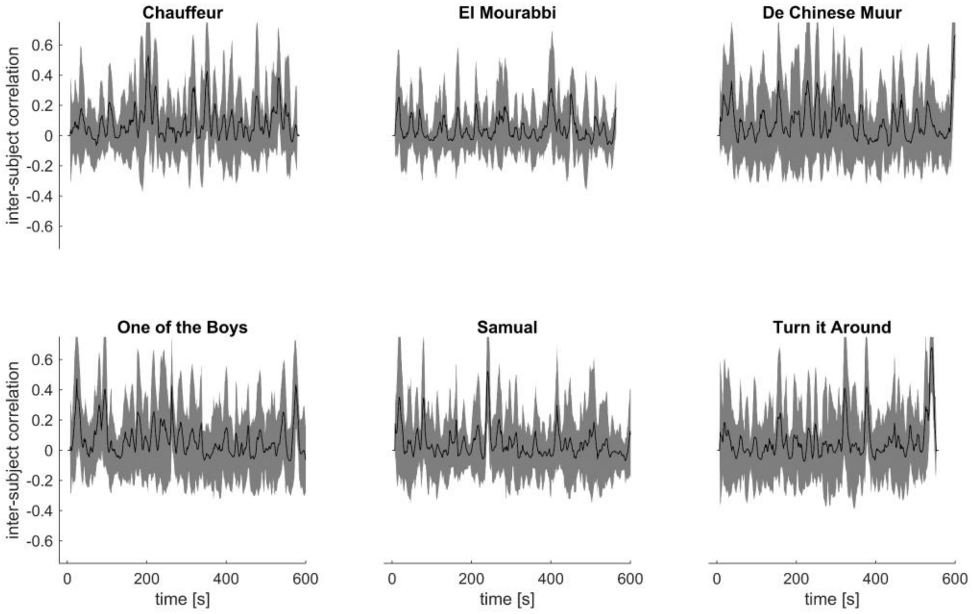

ISC EDA

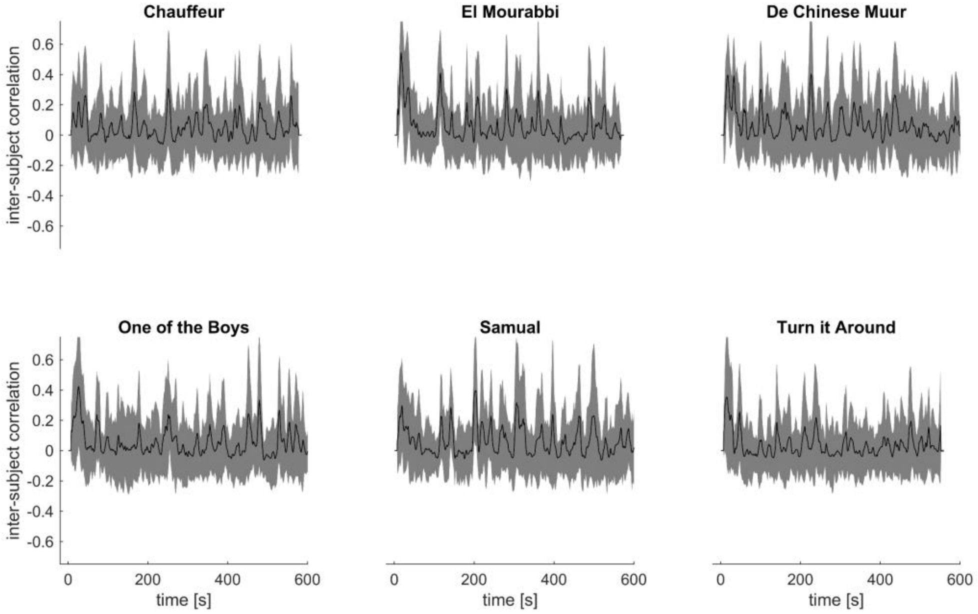

Figure S1. Traces of average inter-subject correlations over time for HR (top) and EDA (bottom) for each of the six movie clips. Shaded areas depict the standard deviation across participants.

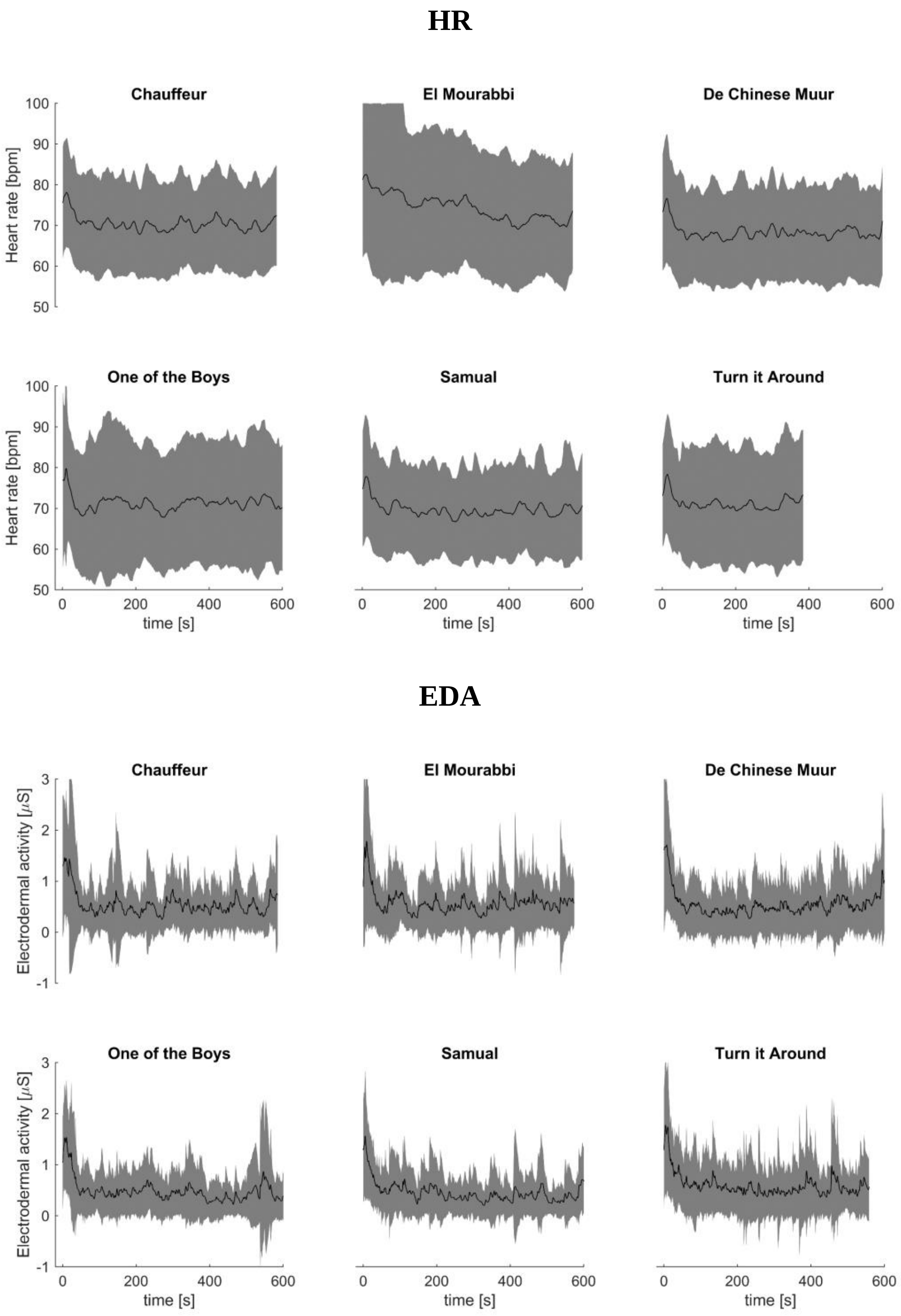

Figure S2. Traces of mean HR (top) and EDA (bottom) responses over time for each of the six movie clips. Shaded areas depict the standard deviation across participants.
